# Training of verbal creativity modulates brain activity in regions associated with language‐ and memory‐related demands

**DOI:** 10.1002/hbm.22901

**Published:** 2015-07-14

**Authors:** Andreas Fink, Mathias Benedek, Karl Koschutnig, Eva Pirker, Elisabeth Berger, Sabrina Meister, Aljoscha C. Neubauer, Ilona Papousek, Elisabeth M. Weiss

**Affiliations:** ^1^ Institute of Psychology BioTechMed Graz, University of Graz, Austria

**Keywords:** divergent thinking, verbal creativity, training, functional magnetic resonance imaging, inferior parietal cortex, middle temporal gyrus

## Abstract

This functional magnetic resonance (fMRI) study was designed to investigate changes in functional patterns of brain activity during creative ideation as a result of a computerized, 3‐week verbal creativity training. The training was composed of various verbal divergent thinking exercises requiring participants to train approximately 20 min per day. Fifty‐three participants were tested three times (psychometric tests and fMRI assessment) with an intertest‐interval of 4 weeks each. Participants were randomly assigned to two different training groups, which received the training time‐delayed: The first training group was trained between the first and the second test, while the second group accomplished the training between the second and the third test session. At the behavioral level, only one training group showed improvements in different facets of verbal creativity right after the training. Yet, functional patterns of brain activity during creative ideation were strikingly similar across both training groups. Whole‐brain voxel‐wise analyses (along with supplementary region of interest analyses) revealed that the training was associated with activity changes in well‐known creativity‐related brain regions such as the left inferior parietal cortex and the left middle temporal gyrus, which have been shown as being particularly sensitive to the originality facet of creativity in previous research. Taken together, this study demonstrates that continuous engagement in a specific complex cognitive task like divergent thinking is associated with reliable changes of activity patterns in relevant brain areas, suggesting more effective search, retrieval, and integration from internal memory representations as a result of the training. *Hum Brain Mapp 36:4104–4115, 2015*. © **2015 The Authors Human Brain Mapping Published by Wiley Periodicals, Inc.**

## INTRODUCTION

In view of the rapidly increasing complexity of the world around us “creativity is more important now than ever before” [Runco, 2004, p. 658] and is even considered as "a useful and effective response to evolutionary changes "[Runco, 2004], since it allows the individual to flexibly respond to the continuously changing conditions around us. As a result, creativity is becoming increasingly attractive in science, and great interest has been devoted to the crucial research question as to how this important ability can be trained.

Research from the psychometric research tradition has indeed revealed some factors that may unfold beneficial effects on creativity, among the most important being positive affect [e.g., Ashby et al., 1999; Baas et al., 2008] and cognitive‐oriented interventions. The latter intend to improve creativity‐related skills by providing specific rules, techniques, or strategies to develop appropriate cognitive skills for the domain at hand [Scott et al., 2004]. This could be realized, for instance, through creative ideation trainings or divergent thinking exercises [Benedek et al., 2006; Coskun, 2005], which aim at stimulating effective search, retrieval, and integration/combination of remote associations related to a given stimulus word. Stevenson et al. [2014], for example, practiced their participants in generating alternative uses for everyday objects over a time period of 2 weeks. Two active control groups were used which both received a training of cognitive processes that are associated with but not directly related to creative ideation (generation of ordinary characteristics of everyday objects, and rule‐switching). Results revealed that all three training conditions yielded training effects on both fluency and originality in the Alternative Uses task (AUT).

At a more basic level, beneficial effects on creative ideation could be achieved via cognitive stimulation in which participants—as is the case in group‐based brainstorming—are confronted with and actively attend to other people's ideas while they are thinking of new ideas [Dugosh and Paulus, 2005; Dugosh et al., 2000]. Fink et al. [2012] found that cognitive stimulation via common or moderately creative ideas was effective in improving verbal creativity, and most strikingly, stimulation effects were also apparent at the level of the brain. Results revealed a widespread creativity‐related neural network mainly including the left middle and superior temporal gyri, along with the right parietal cortex as being particularly sensitive to cognitive stimulation.

Although neuroscientific creativity training studies are rare, there are some recent studies which investigated changes in resting state functional connectivity as a result of verbal divergent thinking interventions. Cousijn et al. [2014] conducted an eight‐session AUT training in a sample of 32 adolescents and in a pretest post‐test design they measured functional resting state connectivity patterns in task‐relevant brain regions such as the bilateral middle temporal gyri (MTG), the medial frontal gyrus, and the left supramarginal gyrus (SMG). They found that at pretest stronger connectivity between the MTG and bilateral postcentral gyri was associated with better divergent thinking performance, but this study revealed no training effects on divergent thinking and resting state functional connectivity; they only report that changes of divergent thinking performance over time were predicted by connectivity between the left SMG and right occipital brain regions. Wei et al. [2014] investigated resting state functional connectivity in relation to creativity in a large sample of participants (*n* = 269), and in a smaller subsample (*n* = 34) they additionally investigated changes in resting state connectivity after cognitive stimulation (via the presentation of external ideas). They found that resting state functional connectivity between the medial prefrontal cortex and the MTG was positively associated with creativity, and most interestingly, they were also able to demonstrate performance increases in originality and increased connectivity between these brain regions after cognitive stimulation.

In this study, we investigated the effects of a comprehensive, cognitive‐oriented verbal creativity training on functional patterns of brain activation during the generation of creative ideas. The employed training (*CreaTrain*) is a computerized, 3‐week verbal creative ideation training composed of various verbal divergent thinking exercises requiring the trainee to generate a broad range of different ideas (e.g., inventing names, finding nicknames, etc). Training effects were investigated in a longitudinal research design in which two groups of participants were trained time‐delayed and tested at three time points with an intertest‐interval of approximately 4 weeks each. At each time point of assessment, functional patterns of brain activity during the performance of well‐known verbal creative ideation tasks were assessed by means of functional magnetic resonance imaging (fMRI), and psychometric tests for the assessment of different facets of creativity were administered. We were particularly interested to see whether the training modulates brain activity in a network of regions that has been associated with creativity in previous research.

## METHOD

### Participants

Sixty volunteers (mostly university students) were recruited for this training study. Five participants quitted participation after the first test session, while two participants had to be excluded from further analyses due to technical problems during fMRI assessment. The final sample comprised 53 participants (26 females, 27 males) in the age range between 19 and 34 years (*M* = 24.04; SD = 2.93) who were tested at three different time points, both with respect to psychometric test variables and with respect to fMRI during the performance of verbal creativity tasks (as described in detail below). The participants were randomly assigned to two training groups: Training group 1 (TG 1; *n* = 24, 11 females) received the training between the first (t1) and the second test session (t2), while the second training group (TG 2; *n* = 29, 15 females) was trained between t2 and the third test session (t3). In using this design (three test sessions and two training groups), we expected the participants of TG1 to show increases in verbal creativity (along with changes in functional patterns of brain activity) from the first to the second test session, while TG2 should show no substantial changes during this time. The latter group was expected to exhibit improvements in verbal creativity from t2 to t3, that is, right after the training. The data of the third test session of the TG1 also allowed examining whether any training effects remain stable over time, while the data from TG2 at t2 provide a test of potential changes that are unrelated to training (e.g., retest effects).

Both training groups did not differ significantly (*P* > 0.05) with respect to sex, age, the Big Five dimensions of personality, and with respect to a proxy measure of intelligence (Wonderlic Personnel Test [WPT]) [Wonderlic, 1999]. All participants were healthy, with no history of substance abuse or other medical, psychiatric or neurological disorder. They were right‐handed (as determined by self‐report), had normal or corrected‐to‐normal vision, gave written informed consent, and received an expense allowance for their participation in the MRI study. The study was approved by the local ethics committee of the University of Graz, Austria.

### Psychometric Tests

#### Pretraining psychometric assessment

Prior to fMRI recording participants were tested with respect to personality, intelligence and verbal creativity. We assessed the Big Five factors of personality (extraversion, agreeableness, conscientiousness, neuroticism, openness to experience) by means of the Neuroticism Extraversion Openness Five Factor Inventory by Costa and McCrae [German adaptation by Borkenau and Ostendorf, 1993]. Participants also completed the WPT, a rough screening instrument for the assessment of intelligence [Wonderlic, 1999]. This test requires the processing of disordered sentences, analogies, number series, word and sentence comparisons, and geometrical figures within a given time period of 12 min. In addition, we administered the verbal imagination subscales of a well‐established German cognitive ability test (“Berliner Intelligenz‐Struktur‐Test,” BIS) [Jäger et al., 1997], to obtain a comprehensive measure of participants' verbal creativity. The employed tasks require flexible idea generation, the availability of manifold and variegated information, richness of imagination and the capability to see different perspectives/variants of objects and problems, and problem‐oriented solutions rather than unsystematic floating of fantasy. Participants worked on four different tasks (with time limits ranging between 2 and 2.5 min) that required them to operate creatively with verbal stimuli (constructing different sentences with given words), respond creatively to given situations, or to generate original uses of everyday objects. In total, the four BIS subtests provide seven scores (four for the assessment of ideational fluency, and three for flexibility). These scores were *z*‐standardized and aggregated into a global measure of verbal creativity, which was then correlated with task performance during fMRI assessment to investigate the validity of the employed creative ideation tasks during imaging.

#### Verbal creativity training

The verbal creative ideation training *CreaTrain* was provided as training software to be installed on the participants' home PC's. It was delivered to the participants with a manual explaining the installation procedure, along with a training schedule. Basically, the training required participants to fluently generate creative/original ideas to a broad range of verbal creativity tasks that were adopted from well‐known psychometric creativity tests such as the Torrance Tests of Creative Thinking [Torrance, 1966], Schoppe's [1975] “Verbaler Kreativitäts‐Test” or the imagination subscales of the “Berliner Intelligenz‐Struktur‐Test” (BIS) [Jäger et al., 1997]. A prototype of this training has already proven to be effective in previous research [Benedek et al., 2006; Fink et al., 2006].

Approximately half of the training exercises explicitly required the participants to be fluent and creative in the verbal domain, such as word completion (e.g., name words that include the syllable “DE”), finding slogans (e.g., slogans for the new product “orange‐icecream”), producing nicknames (e.g., for “coffee”), or generating sentences with three given stimulus words (e.g., “car—fish—book”). The other half of the exercises still used verbal stimuli but required to be fluent and creative in the functional domain. These exercises dealt, for example, with the generation of characteristics of objects and situations (e.g., think of basic features of an “apple”), product improvements (e.g., how could a “bicycle” be improved?), or finding explanations and consequences of given situations (e.g., “what would be the consequences of a new ice age?”). Participants worked at least 2 min on each task and received feedback on training time and the amount of generated ideas. Overall, the training consisted of 144 exercises, which were organized into 18 training modules/units, each taking approximately 20 min to complete [for further details see Benedek et al., 2006]. Participants were instructed to complete ideally six units per week, and to establish comparable training conditions across participants they were requested to exercise not more than three training units per day and to pause not longer than three days. The total training duration was approximately 3 weeks. The records of training data (training time, number of generated ideas per module, etc.) suggest that the participants completed all tasks in time and were fully engaged in the training.

#### Psychometric assessment of training effects (outside the scanner)

To assess behavioral/psychometric training effects, a brief verbal creativity measure, a word fluency scale, and a screening test for the assessment of figural/graphical creativity were administered at each test session (t1, t2, t3). For each of these measures, parallel test versions (items) were available for each time point of assessment.

As a measure of word fluency, two single letters were presented at each session [t1: “F,” “K”; t2: “L,” “S”; t3: “R,” “P”; cf. word fluency test of the “Leistungsprüfsystem LPS by Horn, 1983] and participants were required to generate as many different words beginning with the specified letter as possible. For each letter the time limit was 1 min.

Verbal creativity was assessed by means of verbal idea generation tasks (*creative explanation* task [CE]) [cf. Fink et al., 2007]. In each test session, two situations were presented in short sentences (e.g., “A sound breaks the silence”) and participants were required to write down as many and as original explanations for the situations as possible. Task performance was quantified by means of fluency (i.e., number of ideas) and originality. Originality was scored by means of the top‐3 method reflecting the average rated originality of the three most creative ideas per item, a method that ensures a valid creativity measure beyond mere fluency [Benedek et al., 2013; Jauk et al., 2014]. The three most creative ideas as selected by the participants were evaluated by four raters for creativity (“0” = uncreative, to “3” = very creative), and ratings were averaged across raters and items. A total verbal creativity score was then computed by averaging *z*‐standardized fluency and originality scores of the CE task.

To examine potential transfer effects of the verbal creativity training to the domain of figural creativity, a screening test for the assessment of figural/graphical creativity (“Test zum Schöpferischen Denken – Zeichnerisch,” TSD‐Z) [Urban and Jellen, 2010] was employed. In this test, participants are presented an incomplete picture on a sheet of paper, which is composed of abstract figures and lines, and they are instructed to complete or extend the given fragments as originally as possible. The TSD‐Z is scored with respect to 14 different categories (such as the number of continuations/extensions, number of new added elements, unconventionality, etc.) and provides a total score of participants' creative potential in the figural/graphical creativity domain.

#### Creativity tasks during fMRI assessment

BOLD response was measured during the performance of the AUT, which requires the participants to generate unusual and original uses of given conventional everyday objects (such as “*tin*” or “*umbrella*”). In the control task, participants worked on the Instances Task (IT) [Wallach and Kogan, 1965; Ward, 1968], requiring them to fluently generate conditions or facts that apply to a given adjective (such as “round”). While the AUT could be considered as measure of creative potential (drawing on both the fluency and the originality facet of creativity), the IT merely requires the generation of common and typical facts/examples of given stimuli (without any originality instruction). Both tasks hence considerably differ with respect to their creativity‐related demands, with the AUT being more concerned with the originality facet of creativity. In contrasting functional brain activity patterns during the performance of the AUT and the IT, we are, thus, able to reveal activity patterns that are particularly sensitive to the generation of original/creative ideas.

**Figure 1:**
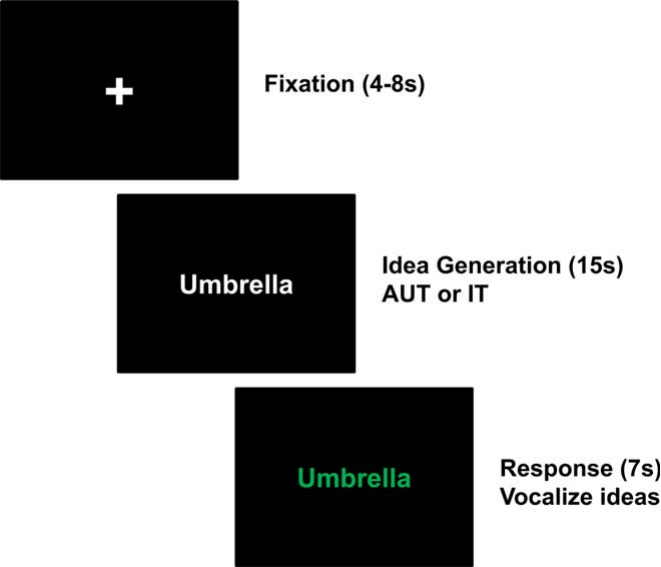
Overview of experimental tasks during fMRI assessment. Each trial started with the presentation of a fixation cross (presentation time jittered between 4 and 8 s). Subsequently, either a noun (AUT) or an adjective (IT) was presented and remained on the screen for 15 s, referred to as idea generation period. Participants were presented 20 items in each task condition (AUT, IT), resulting in a total number of 40 trials. During the idea generation period, participants had to silently think of possible responses to the given stimulus word (either creative/original uses of given objects in the AUT, or facts/conditions/characteristics that apply to given adjectives in the IT) and they were requested not to speak. After the idea generation period the color of the stimulus word changed from white into green, and the participant was now allowed to articulate his or her ideas (Response interval 7s). [Color figure can be viewed in the online issue, which is available at http://wileyonlinelibrary.com.]

Participants were presented 20 stimuli in each task (AUT, IT), resulting in a total number of 40 trials. At each time point a different set of AUT and IT stimuli was used. As shown in Figure 1, each trial started with the presentation of a fixation cross (presentation time jittered between 4 and 8 s). Subsequently, the stimulus word—either a noun (AUT) or an adjective (IT)—was presented and remained on the screen for a time period of 15 s, referred to as the idea generation period. During this phase participants had to silently think of possible responses to the given stimulus (either unconventional/original uses of everyday objects or instances of given adjectives) and they were requested not to speak. After the idea generation period the color of the stimulus word changed from white into green, signaling the participant to articulate his or her ideas. The response interval was 7 s. The oral responses were recorded and transcribed for further analyses. The order of presentation of experimental conditions was randomized. The total time of task presentation was about 20 min and the entire MRI session (involving other imaging sequences) took about 40 min.

Creative task performance during fMRI assessment was quantified via ideational fluency (i.e., number of generated ideas) and originality of ideas in the AUT. The originality rating procedure of the AUT responses was similar as for the psychometric tests outside the scanner. Again four raters evaluated the ideas of the participants on a four‐point rating scale ranging from “0” (“not original at all”) to “3” (“highly original”). The raters received lists of all (nonredundant) responses of the participants in an alphabetical order and were hence blind to the relationship of responses and experimental groups. For each trial we computed a top‐1 score (i.e., rating of only the most creative idea per task, because the task duration was much shorter than in the psychometric test session), which then was averaged across all 20 trials. Inter‐rater agreement was satisfactory (intraclass correlation: 0.75). For further analyses we computed a composite measure of AUT creativity for each time point of assessment by aggregating the *z*‐standardized scores for AUT fluency and AUT originality. This composite measure was significantly (*P* < 0.01) associated with the total score of the full‐length verbal imagination subscales of the BIS, a widely used and proven psychometric measure of verbal creativity (t1: *r* = 0.57, t2: *r* = 0.51, t3: *r* = 0.39). This finding supports the validity of the AUT during fMRI assessment.

### FMRI data acquisition

Imaging was performed on a 3T Siemens Magnetom Skyra tomograph (Siemens Medical Systems, Erlangen, Germany) using a 32‐channel head coil. BOLD‐sensitive T2*‐weighted functional images were acquired using a single shot gradient‐echo EPI pulse sequence (TR = 2.4 s, TE = 30 ms, flip angle = 90°, slice thickness = 3.5 mm, voxel size = 3.5 × 3.5 × 3.5, FoV = 240 mm, 36 axial slices per volume). To record the verbal response of the participants, a MR compatible microphone was used (FOMRI‐III, Optoacoustics Ltd., Moshav Mazor, Israel). Visual stimuli were presented using the Software Presentation (Neurobehavioral Systems, Albany, CA).

### FMRI data analysis

Functional MRI data analysis was performed using SPM8 software (Wellcome Department of Imaging Neuroscience, London, UK). Preprocessing steps included motion correction, slice time acquisition correction, and spatial normalization into the standard space (Montreal Neurological Institute). Finally, the functional data were smoothed using a Gaussian filter of 8 mm. A high‐pass filter with a cut‐off frequency of 1/128 Hz was employed to remove low frequency drifts.

For each participant all three time points were entered into one design matrix and linear *t*‐contrasts between both experimental conditions (AUT > IT; IT > AUT) for each time point (t1, t2, t3) were computed. These contrasts were then entered into a random effects one‐sample *t*‐test for each training group (TG1, TG2) and time point of assessment. All reported activations were corrected for multiple comparisons by means of AlphaSim (http://afni.nimh.nih.gov/afni/doc/manual/AlphaSim) equivalent to *P* < 0.05. Following this approach the smoothness of each contrast was estimated individually and the minimum cluster size ranged between 25 and 28 voxels (uncorrected voxel‐level = *P* < 0.0001, 10,000 iterations).

## RESULTS

### Behavioral Results— Analysis of Training Effects

The efficacy of *CreaTrain* on verbal creativity was analyzed both with respect to AUT performance during fMRI assessment (mean of *z*‐standardized scores of AUT fluency and AUT originality) and with respect to psychometric test performance outside the scanner (mean of *z*‐standardized scores of fluency and originality in the CE task, performance in the word fluency test). For each dependent variable separate repeated measurement ANOVAs with the factors TIME (t1, t2, t3) and experimental GROUP (TG1, TG2) were computed.

**Figure 2:**
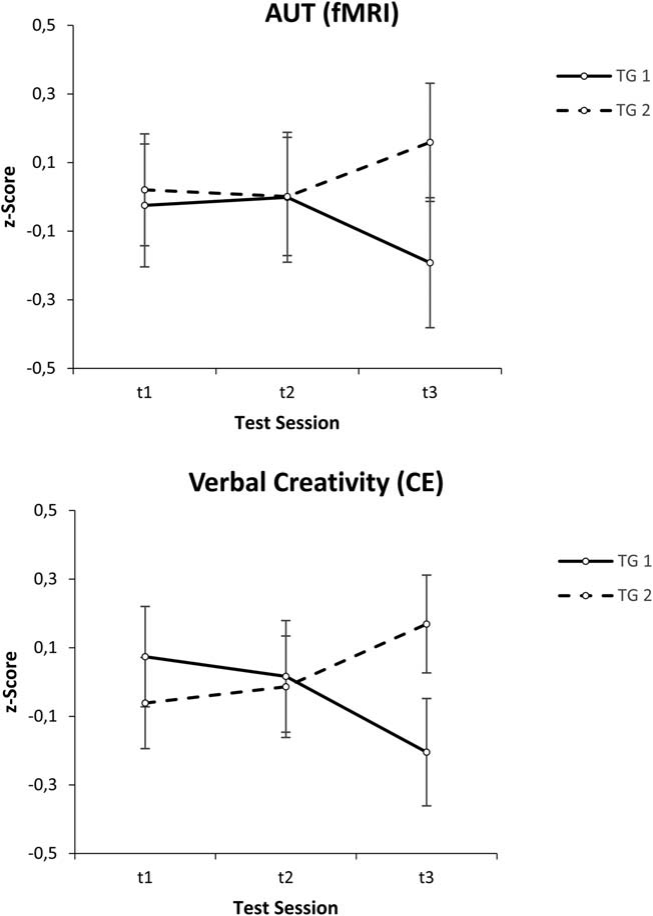
Effects of the creativity training *CreaTrain* on verbal creativity. Task performance during fMRI assessment (upper part) and psychometric test performance outside the scanner (lower part) for both training groups (TG1 and TG2) and the three test sessions (t1, t2, t3). Task performance during fMRI recording refers to a composite measure (mean *z*‐scores) of AUT (alternate uses task) fluency and originality; psychometric test performance (outside the scanner) refers to a composite measure (mean *z*‐scores) of CE (creative explanations) fluency and originality.

The ANOVA for the control task (IT) revealed no significant findings (relevant TIME by GROUP interaction: *F* (2, 102) = 1.64, *P* = 0.20), suggesting that the mere generation of instances to given adjectives was not significantly modulated by the training (see Table 1). The interaction between TIME and GROUP was significant with respect to AUT performance during fMRI assessment, *F* (2, 102) = 3.42, *P* = 0.04, *η^2^p* = 0.06. Subsequent paired *t*‐tests separately for both training groups revealed significant differences only in TG2 from t2 to t3 (*t* (28) = −2.16, *P* = 0.04), suggesting an increase in AUT performance right after the training in this group (see Figure 2A).

**Table 1:** Descriptive statistics (*M*, SD) of performance measures (*z*‐standardized scores) obtained during fMRI assessment (AUT, IT) and outside the scanner (Creative Explanation, CE; Word fluency; Figural creativity)

			t1		t2		t3	
			M	SD	M	SD	M	SD
TG1	AUT		−0.02	0.88	0.00	1.00	−0.19	0.98
	IT		0.05	1.09	0.13	1.12	−0.04	1.08
	CE		0.07	0.64	0.02	0.92	−0.20	0.80
	Word fluency		0.04	1.01	0.28	0.96	0.07	0.98
	Figural creativity		−0.22	1.16	−0.33	1.00	−0.38	1.01
TG2	AUT		0.02	0.88	0.00	0.86	0.16	0.89
	IT		−0.04	0.94	−0.11	0.89	0.03	0.95
	CE		−0.06	0.77	−0.01	0.68	0.17	0.74
	Word fluency		−0.04	1.01	−0.23	0.99	−0.06	1.03
	Figural creativity		0.18	0.83	0.27	0.93	0.31	0.89

The findings for the composite measure of verbal creativity obtained outside the scanner were strikingly similar. The ANOVA again revealed a significant TIME by GROUP interaction (*F* (2, 102) = 4.58, *P* = 0.01, *η^2^p* = 0.08), and as shown in Figure 2B, the overall pattern of this interaction largely matched that observed during fMRI assessment. Paired *t*‐tests revealed a significant difference only in TG2 between t1 and t3 (*t* (28) = −2.22, *P* = 0.04), indicating an increase in verbal creativity in this group. With respect to word fluency, both groups tended to increase their performance right after the training (see Table 1), although, however, the relevant interaction between TIME and GROUP just failed to reached significance (*F* (2, 102) = 2.67, *P* = 0.07, *η^2^p* = 0.05).

Potential transfer effects of the training to the figural creativity domain were investigated by computing a TIME by GROUP ANOVA on the test scores for figural/graphical creativity. This ANOVA only revealed a significant main effect of GROUP (*F* (1, 51) = 6.41, *P* = 0.01, *η^2^p* = 0.11), indicating generally higher figural creativity scores in TG 2 than in TG 1 (see Table 1).

Finally, we examined whether or to which extent individual differences in baseline trait creativity have an impact on the training effects. For this reason, for each creativity measure (AUT, CE, word fluency, figural creativity) 3‐way repeated measurement ANOVAs with TIME and GROUP and the mean BIS verbal creativity score (obtained prior to the training study) as continuously distributed between subjects factor were performed. A significant interaction between TIME, GROUP and BIS creativity was observed only in the CE task, *F* (2, 98) = 6.12, *P* = 0.003, *η^2^p* = 0.11, indicating that in TG1 more creative individuals showed increases in verbal creative task performance right after the training, while less creative individuals showed comparatively strong decreases.

### Neurophysiological Training Effects

#### Contrasts AUT > IT and IT > AUT for TG 1

**Figure 3:**
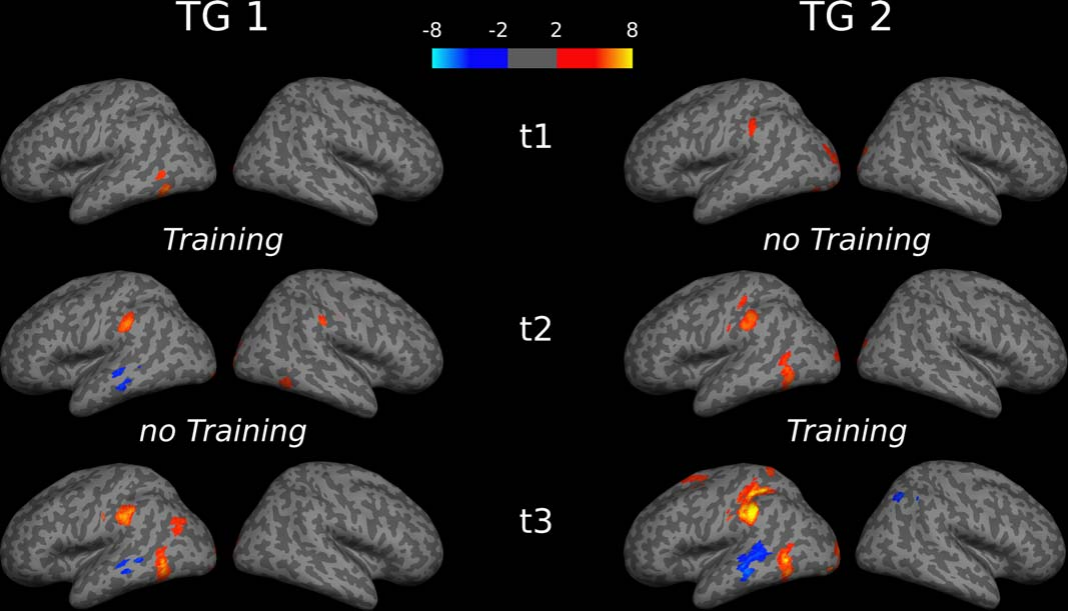
Significant activation clusters of the AUT > IT and IT > AUT contrasts separately for the time points of assessment (t1, t2, t3) and training groups (TG1, TG2). Warm colors (yellow/red) indicate stronger activation in the AUT than in the IT, cold colors (blue) more activation in IT vs. AUT. [Color figure can be viewed in the online issue, which is available at http://wileyonlinelibrary.com.]

Prior to the training (t1), the AUT was associated with stronger activation than the IT in a left‐lateralized brain network involving the calcarine sulcus and regions of the inferior temporal and occipital gyri (see Table 2 and Fig. 3). Analyses revealed no brain regions at t1 that were stronger activated in IT vs. AUT. Immediately after the training, at t2, the AUT vs. IT contrast yielded a comparatively large activation cluster in the left SMG and the left inferior parietal cortex, and a widespread pattern of activation in the visual cortex, also including regions of the right hemisphere. Additionally, at t2 a significant activation cluster was found in which the IT evoked stronger activation than the AUT, including the left middle temporal gyrus (MTG). A quite similar, although more pronounced, pattern of activation was apparent at t3: The AUT (vs. IT) again yielded stronger activation in the bilateral visual cortex, along with activation in posterior parts of the left inferior and middle temporal gyri (also including small portions of the inferior occipital gyrus), and the left IPC (including portions of the supramarginal and angular gyri), whereas the IT again showed stronger activation in the left MTG (see Table 2, Fig. 3).

**Table 2:** Significant activation clusters of the AUT > IT and IT > AUT contrasts separately for the time points of assessment (T1, T2, T3) and training groups (TG1, TG2)

**Time of assessment**	**MNI (Peak)**	**k**	**t**	**Brain area (AAL)**
**TG 1**				
**T1**	−6 −100 −2	93	5.96	L calcarine
	−54 −70 −8	55	6.47	L inf temporal, L inf occipital
**T2**	18 −94 −2	116	6.56	R calcarine, R lingual
	−15 −100 −8	104	6.70	L calcarine, L lingual
	−63 −28 40	79	6.27	L supramarginal, L inf parietal
	51 −55 −8	31	5.38	R inf temporal
	63 −22 40	29	5.65	R supramarginal
	−63 −28 −8	42	−4.95	L mid temporal
**T3**	−12 −94 −5	286	10.52	L lingual, L calcarine
	15 −85 −11	135	8.24	R lingual, R calcarine
	−57 −28 34	131	6.89	L supramarginal, L inf parietal
	−51 −61 −2	95	6.92	L inf/mid temporal
	−42 −79 34	38	5.19	L angular, L mid occipital
	−60 −37 −2	29	−5.27	L mid temporal
**TG 2**				
**T1**	15 −94 −2	593	7.77	R/L calcarine, R/L lingual, L inf/mid occipital
	−57 −31 40	30	4.84	L supramarginal, inf parietal
**T2**	15 −94 1	195	6.63	R calcarine, R lingual
	−60 −25 31	160	5.82	L supramarginal, L inf parietal
	−15 −97 −2	79	5.92	L mid occipital, L calcarine
	−48 −61 −2	75	5.83	L mid/inf temporal
**T3**	−54 −28 37	413	9.07	L inf parietal, L supramarginal, L postcentral
	−12 −97 −2	188	8.19	L mid/inf occipital, L calcarine
	18 −88 −5	187	9.12	R calcarine, R lingual
	−54 −64 −2	108	7.90	L mid/inf temporal
	−12 17 67	73	6.07	L sup/mid frontal
	−30 −49 67	54	5.36	L sup parietal, L Precuneus
	−63 −28 −2	96	−5.96	L mid temporal
	45 −61 55	30	−5.35	R inf parietal, R angular

Positive *t*‐values indicate stronger activation in the AUT than in the IT, negative values more activation in IT vs. AUT.

AAL = Automated Anatomical Labeling, L = left hemisphere, R = right hemisphere, inf = inferior, sup = superior, mid = middle.

#### Contrasts AUT > IT and IT > AUT for TG 2

As was the case in the TG1, also in the TG2 activation was primarily restricted to the visual cortex (more activation in the AUT vs. IT) prior to the training (at t1), apart from a small activation cluster (*k* = 30) involving the left SMG and the left IPC (Table 2). Likewise, there were no brain regions at t1 that were more activated in the control task (IT) than the AUT. At t2, the AUT again revealed stronger activation than the IT in bilateral occipital brain regions and in the left SMG and IPC, now also in the left inferior and middle temporal gyri. No regions were associated with stronger activation in the IT than in the AUT at t2. After the training (t3), a more distinct pattern of activation was observed: Along with activation in the bilateral visual cortex, the AUT was associated with stronger activation in a left‐lateralized brain network primarily involving the IPC, SMG, regions of the superior/middle frontal gyri, the left posterior MTG and inferior temporal gyrus (also including small portions of the inferior occipital gyrus). Only after the training there were also brain regions that were more strongly activated in the IT than in the AUT, including regions of the left MTG and right IPC (see Table 2, Fig. 3).

#### Time‐related changes of brain activity in functionally defined regions of interest

To provide a formal test of the neurophysiological effects of the creativity training, time‐related changes of the contrast estimates (AUT > IT) in functionally defined ROIs were investigated. The ROIs were defined on the basis of a combined mask including all significantly (de‐)activated voxels as revealed by the whole brain AUT vs. IT contrasts across all time points and both groups, hence representing relevant brain areas associated with AUT task performance. This procedure resulted in 10 clusters, for which separate TIME (t1, t2, t3) by GROUP (TG1, TG2) repeated measurements ANVOAs were performed.

**Figure 4:**
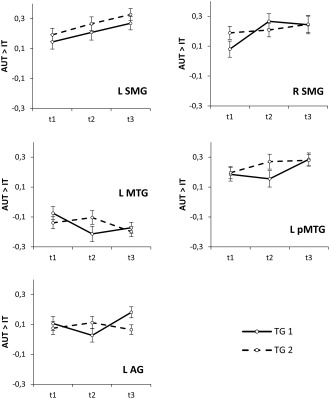
Time‐related changes of brain activity patterns (contrast estimates of the AUT vs. IT contrasts) in functionally defined ROI. Significant effects involving TIME of assessment (including trends toward significance) were found in the bilateral supramarginal gyri (SMG), the left middle temporal gyrus (MTG), the left posterior middle temporal gyrus (pMTG) and the left AG.

The ANOVAs yielded significant main effects of TIME for the SMG bilaterally (left: *F* (2, 102) = 4.68, *P* = 0.01, *η^2^p* = 0.08; right: *F* (2, 102) = 4.28, *P* = 0.02, *η^2^p* = 0.08), suggesting general increases in AUT (relative to IT) activation as a function of time. Inspection of Figure 4 reveals that in the right SMG the significant TIME effect seems to be driven by TG1, which showed comparatively strong activation increases right after the training (i.e., from t1 to t2; *t*(23) = −3.16, *P* = 0.004). The relevant interaction between TIME and GROUP, however, was not significant (*F* (2, 102) = 2.03, *P* = 0.14, *η^2^p* = 0.04). In addition to the significant TIME effects in the bilateral SMG, brain activation in the left posterior MTG tended to increase from t1 to t3 (TIME: *F* (2, 102) = 2.69, *P* = 0.07, *η^2^p* = 0.05).

As was to be expected on the basis of the whole brain voxel‐wise analyses, both groups tended to decrease AUT (relative to IT) activation in the left MTG (see Fig. 4) right after the training, although the relevant interaction between TIME and GROUP just failed to reach statistical significance (*F* (2, 102) = 2.64, *P* = 0.08, *η^2^p* = 0.05). A significant TIME by GROUP interaction was found in the left AG (*F* (2, 102) = 3.77, *P* = 0.03, *η^2^p* = 0.07, suggesting comparatively strong activation increases in TG1 from t2 to t3 (see Fig. 4). Finally, the ROI analyses suggested that the TG2 exhibited stronger activation than the TG1 in superior/middle frontal brain regions (*F* (1, 51) = 4.19, *P* = 0.046, *η^2^p* = 0.08), and a tendency toward more pronounced AUT deactivations in the right inferior parietal cortex (*F* (1, 51) = 3.24, *P* = 0.08, *η^2^p* = 0.06).

To assess whether or to which extent any time‐related changes in brain activation patterns were influenced by the success of training, for each ROI TIME (t1, t2, t3) by GROUP (TG1, TG2) ANCOVAs were computed in additionally considering AUT performance right after the training (TG1: t2; TG2: t3) as continuously distributed between subjects factor. The ANCOVAs yielded no significant interactions involving AUT performance. There was, however, a significant main effect of AUT performance in the left pMTG (*F* (1, 49) = 4.76, *P* = 0.03, *η^2^p* = 0.09), and a trend toward significance in the left SMG (*F* (1, 49) = 3.03, *P* = 0.09, *η^2^p* = 0.06), in each case indicating a positive association between brain activation and AUT performance at the post‐test.

## DISCUSSION

Behavioral findings revealed that in TG2 3 weeks of extensive verbal creativity training were effective in improving verbal creative ideation. This was reflected in performance data during fMRI assessment and psychometric test data obtained outside the scanner, which both showed significant increases in different facets of verbal creativity right after the training. In TG1 no training‐related changes in verbal creative ideation were found, aside from weak increases in word fluency (see Table 1) and performance improvements in the CE task in verbally more creative individuals. Despite this unclear picture at the behavioral level, functional patterns of brain activity were strikingly similar across both groups. Whole brain voxel‐wise analyses revealed that prior to the training both training groups exhibited stronger activation in the AUT relative to the control task (IT) mainly in the visual cortex. After the training, the generation of creative ideas was associated with a much more distinct pattern of activation (see Fig. 3). This was on the one hand particularly apparent in additional or more pronounced AUT (vs. IT) activation in regions of the left IPC (mainly the left SMG) and posterior portions of the inferior and middle temporal gyri after the training. On the other hand, only after the training the AUT was associated with lower activation than the IT in the left MTG, both in the TG1 and in the TG2. Subsequent ROI analyses suggested general time‐related increases in activation in the bilateral SMG and in the left posterior MTG from t1 and t3 across both training groups, while the effects in more anterior portions of the left MTG appear to be more specific to the training.

The MTG has been associated with declarative memory demands [Squire et al., 2004), and is known as part of the semantic system of the brain, responsible for storage and retrieval of semantic information [Binder et al., 2009]. The lower activation of the left MTG in the AUT as compared to the IT could, thus, hint at reduced retrieval from declarative memory when more effective (less retrieval‐based) [cf. Gilhooly et al., 2007] generation strategies are employed after the training. An interesting finding in this context, however, was that creative idea generation was at the same time associated with increasingly stronger activation in more posterior regions of the left MTG, also involving the posterior inferior temporal gyrus (ITG) and smaller portions of the inferior occipital gyrus (see Figs. 3, 4). Notably, participants who exhibited higher scores in the AUT right after the training, generally showed stronger activation in this region. Taken together these findings strongly strengthen the idea that the generation of creative ideas may rely on functionally different areas within the left MTG. Whitney et al. [2011] argued that successful semantic cognition involves on the one hand semantic representations or the semantic store itself, and on the hand executive semantic control processes that shape semantic retrieval in a manner that is appropriate for a specific context or task. They used repetitive transcranial magnetic stimulation (TMS) to disrupt processing within the posterior MTG and the inferior frontal gyrus, which resulted in a disruption of tasks involving high semantic control demands, while more automatic semantic decisions were not affected by TMS. Translated to our findings a possible interpretation would be that, as reflected in the lower AUT (vs. IT) activation in more anterior regions of the left MTG, mere representational task requirements or the mere retrieval of semantic information became more efficient (or were replaced by more effective strategies requiring higher semantic control, respectively) as a result of the training. On the other hand, as reflected by activity increases in the left posterior MTG, the demands on effective semantic control necessary to integrate and effectively combine available semantic information to produce novelty increased.

In line with this idea, Fink et al. [2012] found that when participants were cognitively stimulated with ideas of other people, regardless of the quality of ideas (moderately creative or highly creative), the generation of creative ideas was associated with stronger activation in the left posterior MTG (as opposed to the stimulation with meaningless pseudowords), which could most likely reflect higher demands on semantic information processing as a result of cognitive stimulation with external ideas. When idea generation after the stimulation with highly original ideas (e.g., use of a tin as mini‐biotope or cocktail shaker) was compared to that after the stimulation with moderately creative ideas (e.g., use a flower vase as water pitcher or for decoration), the former additionally evoked activation in the left hippocampus, and as found in this study, also in the left ITG and in the left inferior occipital gyrus. It seems that the more idea generation is targeted toward originality, the deeper semantic processing may be required, as possibly reflected in the additional recruitment of this left posterior temporo‐occipital network. The observed activations in these regions after the training in this study could hence reflect increased effectivity in the retrieval of more “enriched” or wide‐ranging internal memory presentations during the generation of creative ideas. We may therefore conclude that along with the left IPC, the left posterior MTG may be involved in more demanding/sophisticated semantic processes, thus, facilitating effective creative idea generation, which involves the construction of novel representations based on available episodic memory [Schacter et al., 2007].

Another important finding of this study was that continuous engagement in creative ideation tasks was associated with increasingly stronger activation in the left and to some minor extent also in the right supramarginal gyri. Interestingly, those participants who showed better AUT performance right after the training generally tended to display stronger activation in the left SMG. The left IPC (including the left SMG and left angular gyrus) has consistently been found to show stronger activation during the generation of creative/original ideas (AUT) than during the performance of tasks involving lower creativity‐related demands [Cousijn et al., 2014; Fink et al., 2009, 2010, 2014b; Kleibeuker et al., 2013; see also Abraham et al., 2012; Bechtereva et al., 2004]. Imaging studies have generally associated the left IPC with directing attention to internal knowledge representations [e.g., Kahn et al., 2004; Shannon and Brucker, 2004], mental simulation, imagining or future thought [Schacter et al., 2007], processes which might be also crucially implicated in tasks involving divergent thinking demands. The IPC is known as being among the core regions of the semantic network of the brain [Binder et al., 2009], and given its location adjacent to multiple multisensory processing streams, it may have a particular role in high level integrative processes that constitute a key component in various cognitive demands [Binder and Desai, 2011; Binder et al., 2009]. Benedek et al., [2014a] recently reported that the generation of genuinely new ideas, as opposed to the retrieval of old (i.e., known) ideas was associated with stronger activation in the left IPC including the SMG. It was shown that the retrieval of unusual but known ideas is a common initial strategy in creative idea generation, which however is not effective for generating highly creative ideas [Gilhooly et al., 2007]. It hence seems possible that the creativity training helped to use more effective strategies for the generation of novel ideas rather than less effective retrieval‐based strategies. Similarly, there is also evidence that the generation of figurative language (i.e., producing creative metaphors vs. synonyms) is associated with stronger activation of the left angular gyrus [Benedek et al., 2014b]. It, thus, appears that regions of the left IPC—as it seems to be the case in the left posterior MTG—are particularly concerned when tasks draw on the originality facet of creativity, that is, when participants are required to effectively retrieve, combine, and integrate remote associations to construct novel representations.

Besides the effects in the left IPC and the left MTG, creative ideation was generally also strongly associated with brain activity in the visual cortex. At first glance, these effects appear to be somewhat puzzling since the majority of studies highlighted the role of prefrontal brain regions as being particularly relevant for different creativity‐related task demands [Arden et al., 2010; Dietrich and Kanso, 2010; Gonen‐Yaacovi et al., 2013]. However, despite the prevailing role of the prefrontal cortex in creativity there is increasing evidence that the posterior cortex has an important role in creativity as well. The visual cortex has been found to be involved in visual mental imagery [e.g., Kosslyn et al., 2001]. Similarly Aziz‐Zadeh et al. [2012] found stronger activation during visuospatial creativity tasks relative to a control task in regions of the left lateral occipital cortex, also hinting at a potentially important role of occipital brain regions in mental imagination. A similar picture emerged in the verbal creativity domain. Regions of the primary visual cortex were found to be active during creative vs. uncreative story generation [Howard‐Jones et al., 2005]. Andreasen and Ramchandran [2012] found that regions of the visual cortex such as the lingual gyrus and the cuneus were implicated in the performance of a word association task, which they attributed to mental imagery processes involved in the processing of verbal tasks. The prominent role of posterior brain regions in verbal creativity has also been revealed by EEG studies on creativity which consistently found alpha power increases during verbal creative ideation (relative to rest) at posterior cortical sites [Fink and Benedek, 2014]. Nicely in line with this, a recent structural imaging study [Fink et al., 2014a] found evidence that different facets of verbal creativity (among others originality in the AUT) were significantly and positively associated with gray matter density in occipital brain regions such as the cuneus.

Among the most important strengths of this study is the fact that we applied a longitudinal research design including three time points of assessment and two training groups, which received the training time‐delayed. Moreover, brain activation was measured during performance of well‐known creative ideation tasks, which hence extends findings from previous resting‐state training or intervention studies. Finally, we also recorded oral responses during performance of the scanner tasks, which enabled us to monitor and evaluate task performance (by means of fluency and originality) and relate it to brain activation. The employed longitudinal research design does also provide some information on the stability of fMRI effects. It was found that the activation patterns of the AUT vs. IT contrasts were fairly stable: On the one hand across groups, when we compare the activation patterns of the TG1 and TG2 at t1 (prior to the training), and on the other hand as a function of time, when we look at the TG2 which showed similar activation patterns at t1 and t2, or at the TG1 which showed quite similar activation at t2 and t3. Generally, the activation patterns of the TG2 were somewhat more pronounced than those of the TG1. Also, only the TG2 showed significant improvements in behavioral measures of verbal creativity. Supplemental analyses with the untransformed fluency and originality scores indicated that both measures showed decreases between t1 and t2 in both trainings groups, possibly indicating that the second time point of assessment was generally more challenging or exhausting for the participants. Another reason for failing significant behavioral training effects in TG1 may also include the different time points of the training (in the TG1 the training was realized with end of academic term, while the TG2 was trained during vacation time). In addition to this, we should also pay attention to the fact that the assessment of reliable and valid behavioral performance indicators during imaging is per se highly challenging, not least due to the fact that participants are required—unlike in the natural environment—to be creative while lying supine in the scanner. In light of this unclear picture of behavioral results in the first training group, it seems even more striking that both training groups showed a highly similar pattern of results at the level of the brain.

Finally, it is noteworthy that this study revealed fMRI effects specifically in a network of brain regions that was previously shown as being particularly relevant for verbal creative thinking demands [such as the left IPC and the left MTG: e.g., Benedek et al., 2014a,b; Fink et al., 2010, 2012; Kleibeuker et al., 2013; Wei et al., 2014; see also Jung et al., 2013]. This study hence provides independent evidence on the relevance of these brain regions for creative thought by means of a longitudinal training study approach.
